# Associations between HLA Class I alleles and the prevalence of nasopharyngeal carcinoma (NPC) among Tunisians

**DOI:** 10.1186/1479-5876-5-22

**Published:** 2007-05-04

**Authors:** Xin Li, Nahla Ghandri, Daniela Piancatelli, Sharon Adams, Deborah Chen, Fu-Meei Robbins, Ena Wang, Alessandro Monaco, Silvia Selleri, Noureddine Bouaouina, David Stroncek, Domenico Adorno, Lotfi Chouchane, Francesco M Marincola

**Affiliations:** 1 Immunogenetics Section, Department of Transfusion Medicine, Clinical Center, National Institutes of Health, Bethesda, MD, 20892, USA; 2 Laboratory of Molecular Immunology and Oncology, Faculty of Medicine of Monastir, Monastir, Tunisia; 3 CNR, Institute for Organ Transplant and Immunocytology, L'Aquila, Italy

## Abstract

The high prevalence of nasopharyngeal cancer (NPC) in Southern Asia and Mediterranean Northern Africa suggests genetic predisposition among other factors. While Human Leukocyte Antigen (HLA) haplotypes have been conclusively associated with NPC predisposition in Asians, Northern African Maghrebians have been less intensely studied. However, low resolution serological methods identified weak positive associations with HLA-B5, B13 and B18 and a negative with HLA-B14. Using sequence based typing (SBT), we performed a direct comparison of HLA class I frequencies in a cohort of 136 Tunisian patients with NPC matched for gender, age and geographical residence to 148 normal Tunisians. The bimodal age distribution of NPC in Maghrebians was also taken into account. HLA frequencies in normal Tunisians were also compared with those of Northern Moroccan Berbers (ME) to evaluate whether the Tunisian population in this study could be considered representative of other Maghrebian populations. HLA-B14 and -Cw08 were negatively associated with NPC (odd ratio = 0.09 and 0.18 respectively, Fisher p_2_-value = 0.0001 and = 0.003). Moreover, positive associations were observed for HLA-B-18, -B51 (split of -B5) and -B57 (p_2_-value < 0.025 in all) confirming previous findings in Maghrebs. The HLA-B14/Cw*08 haplotype frequency (HF) was 0.007 in NPC patients compared to 0.057 in both Tunisian (OR = 0.12; p_2_-value = 0.001) and Moroccan controls. This study confirms several previous associations noted by serologic typing between HLA class I alleles and the prevalence of NPC in Maghrebians populations. In addition, we identified a putative haplotype rare in Tunisian patients with NPC that may serve as a genetic marker for further susceptibility studies.

## Background

The prevalence of Epstein-Barr virus (EBV)-associated anaplastic nasopharyngeal carcinoma (NPC) might be partially dependent upon genetic background affecting predominantly certain ethnic groups^1^. Among them, some regions of Southern Asia suffer about a 100-fold higher prevalence compared with populations not at risk and this high prevalence is maintained upon immigration to Western Countries such as in the U.S. Chinese population Northern African populations (Algerians, Moroccans and Tunisians) referred to as Maghrebians also suffer increased prevalence of NPC which persists upon migration to low risk areas [1,2] Although heterogeneity of viral strains, dietary, environmental and socio-economical variables have been implicated as predisposing factors, it is likely that genetic traits play a significant role at least in Asians [3,4].

Genetic predisposition of NPC is paralleled by a strong associations with some HLA class I alleles which have been extensively studied in the Asian population. Of them, HLA-A2, -B14, -B46 and -B58 predispose to the disease while HLA-A11 exercises a protective effect in Asians [5-12] Moreover, a meta-analysis confirmed a consistent association between HLA-A2, A11, B14, B46 and NPC [13]. With few exceptions [10,11]., most studies employed serologic HLA typing methods that cannot discriminate alleles within large serologic families [14]. Such discrimination, however, is critical since different ethnic groups display significant differences in frequency of HLA alleles within a serologic family with different capacity to bind and present epitopes that may be relevant to the immune defense against EBV-coded or other NPC-associated antigens. For instance, the HLA-A2 serologic family includes about 100 alleles [15] whose distribution is different among ethnic groups with HLA-A*0201 (~90%) and HLA-A*0205 (~5%) predominant among Caucasian and HLA-A*0203 (~20%) -A*0206 (~15%) and -A*0207 (~40%) in Asians [16-19] Interestingly, sequence based typing (SBT) in Taiwanese identified a positive association between HLA-A*0207 (but not other HLA-A2 subtypes) and NPC [10]. This association was particularly strong when the observation was extended to the HLA-A*0207/B*4601 haplotype. Since, chromosome 6p, in particular 6p21-23, is characterized by unusually high gene density harboring gene clusters coding for several protein families besides HLA molecules [20], it remains unclear whether NPC/HLA associations reflect distinct antigen presentation potentials of various alleles or rather represent a marker for a susceptibility/protection locus in strong linkage disequilibrium with the HLA region [21].

Maghrebian populations have been less intensely studied at high-resolution SBT. Serological analyses reported positive associations between NPC and HLA: these included increased frequency of HLA-B5 in Algerians (38.2% *vs *24.4%) [22], HLA-B13 in Tunisians (15.5% vs 4%) [23] and HLA-B18 in Moroccans, of which only the last was significant after correction for number of specificities tested (relative risk = 4.14) [24]. In 1983, Heirat P *et al*. [22] reported in a cohort of patients with NPC from Algeria a lower frequency of HLA-Aw33 (3.9% vs 16.8%), -B14 (1.3% vs 16%) and -DR4 (13.2% vs 29.1%) in NPC compared with matched controls. After correction for the number of specificities tested, these differences were not statistically significant and were not pursued further. Subsequently, HLA-A*3301 and HLA-B*1402 were found to belong to the same ancestral haplotype [25]. Further studies done in other Maghrebians did not reproduce these findings. Independently, Mokni-Baizig N *et al*. [23] noted that the HLA-A23 allele was absent in NPC patients compared to an 18% frequency in non tumor-bearing Tunisians. This is interesting because HLA-A23 is part of an African extended haplotype that includes a -B14 allele (HLA-A*2301/HLA-B*1403) [26]. Moreover, Dardari R et al. [24] reported a significantly lower frequency of HLA-A9 serologic family alleles in NPC patients compared with controls. Since the HLA-A9 serologic family includes HLA-A23, this observation suggests that the reduced frequency of HLA-A9 reflects a lower frequency of the HLA-A*2301/HLA-B*1403 haplotype. Thus, previous studies in Maghrebians suggest that HLA-B14 and HLA-A alleles linked to the HLA-B14 serological family may be under-represented in patients with NPC compared with normal controls. Conversely, HLA-B5, -B13 and -B18 could be associated with increased risk of NPC.

Molecular typing provides enhanced discrimination for population studies and large data bases have accumulated in African populations including Maghrebians [25-29] These studies, however, did not include Tunisians (TU). Therefore, the aim of this study was to 1) establish a high resolution SBT data base in normal Tunisians matched for gender, age and geographical residence to a similar cohort of NPC patients; 2) compare HLA allele and haplotype frequencies between normal healthy Tunisians and the previously characterized Northern Moroccan Berber population (ME) [28] to provide a broader frame for the interpretation of the results from the Tunisian study; 3) test the validity of the reported decreased prevalence of HLA-B14 and increased prevalence of HLA-B5, -B13 and -B18 in patients with NPC based on SBT-derived, high resolution information; 4) Find novel alleles and haplotypes associated with NPC in Tunisian that may serve as putative genetic markers for further susceptibility studies.

## Materials and methods

### Study population

147 Patients with undifferentiated nasopharyngeal carcinoma were recruited from the Department of Radiation Oncology of Sousse Hospital, between 1991 and 2004. The patients with nasopharyngeal cancer had a mean age of 41.5 ± 16 years. The clinical stages ranged from II to IV (TNM classification, 1987). The diagnosis of cancer was confirmed by histopathology analyses. The histology was undifferentiated carcinoma (type III, WHO classification) in all cases [30,31].

Patients were age and sex matched with 150 control subjects and selected from the same population living in the middle coast of Tunisia sharing the same environmental conditions. Control subjects having a mean age of 40 ± 12.5 years were healthy blood donors having no evidence of any personal or family history of cancer (or other serious illness). In order to respect the Hardy-Weinberg conditions highly stringent criteria were used for recruitment. To avoid any consanguinity bias, both patients and control groups were formed with unrelated individuals.

The control Tunisian population data were also compared to an available database of SBT typing performed in a population from North Morocco [28] to provide a frame of reference for this study in the context of other previously studied Maghrebian populations.

### Specimen collection, preparation and HLA genotyping

Blood samples were obtained from all subjects consenting to the collection of blood, [0] through venipuncture and processed for DNA preparation. Extracted DNA was shipped to the Department of Transfusion Medicine, Clinical Center; National Institutes of Health where the genotyping was performed. SBT of HLA class I loci was performed as previously described [32]. The primary PCR amplification reaction produced a 1.5 kb amplicon encompassing exon 1 through intron 4 of the HLA class I locus. All reagents necessary for primary amplification and sequencing were included in the HLA-A, HLA-B and HLA-C allele SEQR Sequenced Based Typing Kits (Abbott Diagnositics, Abbott Park, IL U.S.A.). After the primary amplification PCR products were purified from excess primers, dNTPs and genomic DNA using ExoSAP-IT (American Life Science, Cleveland, OH, U.S.A.). Each template was sequenced in the forward and reverse orientation for exon 2, exon 3 and exon 4 according to protocols supplied with the SBT kits. Excess dye terminators were removed from the sequencing products utilizing an ethanol precipitation method with absolute ethanol. The reaction products were reconstituted with 15 μl of Hi-Di™Formamide (PE Applied Biosystems/Perkin-Elmer, Foster City, CA, U.S.A.) and analyzed on the ABI Prism* 3700 DNA Analyzer with Dye Set file: Z and mobility file: DT3700POP6 [ET].

### Statistical analysis

SBT information was used to compare our data base with recent reports on the prevalence of HLA alleles and haplotypes in African populations [25-29] The Hardy-Weinberg equilibrium was tested using the Guo and Thomson method [33], and gene diversity was estimated by maximum likelihood at each locus. Haplotype frequencies (HF) were estimated using an expectation-maximisation (EM) algorithm for multi-locus genotypic data when the gametic phase is not known employing the Arlequin population genetic software v3.01 [34,35] The Ewens-Watterson homozygosity test [36,37] was used to examine the presence of selective forces influencing allelic diversity at each locus (balancing selection or directional selection). The significance was calculated by using the Ewens-Watterson test and Slatkin exact test [38,39]. HLA class I allele frequencies between Tunisians and Moroccans were compared using a χ^2 ^analysis with Bonferroni correction. HLA class I (haplotipic) genetic diversity between the 2 populations was assessed by computing Fst genetic distance and the exact test of population differentiation using Arlequin. The most frequent 2 loci (Cw-B, A-Cw, A-B) and class I (A,-Cw,-B) haplotypes were described. Linkage disequilibrium (D) and relative D (D') between two alleles at two different loci and their level of significance (p) for 2 × 2 comparisons were calculated [40].

Comparisons between NPC and controls were performed first at the low resolution (two digit discrimination level) to compare our results with previously reported information based on low resolution serological typing. For this descriptive analysis data were simply compared using a 2 × 2 comparison without correction for number of tests. Further, SBT based comparisons between NPC and control populations were performed using allele frequencies and haplotypes calculated with the statistical tests discussed in the previous paragraph.

## Results

### HLA frequencies among non-tumor bearing healthy Tunisians (TU)

To our knowledge no reports are available on SBT-defined HLA class I frequencies among healthy Tunisians. However, extensive information exists on other African/Maghrebian populations [25-29] We, therefore, first analyzed HLA class I frequencies in Tunisian in the context of available information in other Maghrebian populations. In particular, we performed a direct comparison with Piancatelli D. et al [28] SBT data on Moroccans.

**Figure 1:**
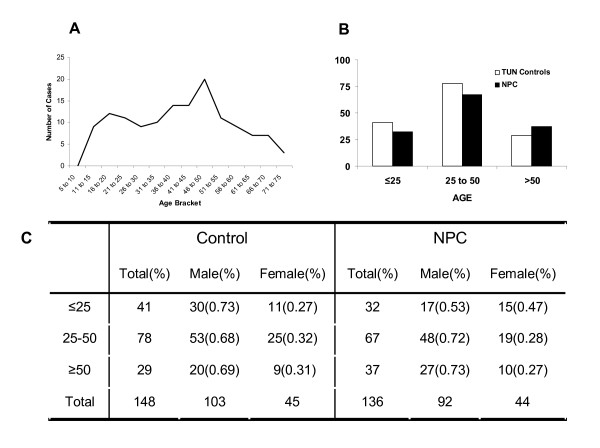
A: age distribution of the 136 patients with NPC whose DNA was a sufficient quality to yields informative typing results. Age is shown in 5 years brackets to underline the bi-modal distribution of NPC prevalence in Tunisian reflected by the population studied. B: Age distribution of patients with NPC (black bars) and normal TU controls (white bars) separated according to recognized brackets of prevalence: precocious NPC (10 to 25 years), middle age NPC (26 to 50) and late onset NPC (> 50 years of age) (lower panel). C: age distribution of patients with NPC and normal TU controls according to age and gender.

Of the 150 samples obtained from normal control subjects only 147 HLA-A, 148 HLA-B and 147 HLA-Cw locus typings could be performed due to insufficient quantity or quality of the recovered genomic DNA in the remaining cases. Because of the recognized bimodal age distribution of NPC in Tunisia an effort was made to collect DNA representative of all age groups in the control population (Figure 1). The allele and haplotype frequencies for the HLA class I loci in the Tunisian population compared to Moroccan are reported in Tables 1, 2, 3. The Tunisian population was in Hardy-Weinberg equilibrium for all loci (HLA-A, p = 0.568 ± 0.000; HLA-B, p = 0.505 ± 0.000; HLA-Cw, p = 0.297 ± 0.000); the allelic distribution showed a very high level of gene diversity: HLA-A 0.94; HLA-B 0.96 and HLA-Cw 0.90. In the Moroccan population, these values were slightly lower for HLA-A (0.92) and HLA-B (0.95), and not different for HLA-Cw. Population comparisons showed that the two populations were not significantly different (Fst p value: 0.189) and no statistically significant differences were observed in allele frequencies between the two populations (Table 1).

**Table 1:** HLA-A, -B and -Cw allele frequencies in ME and TU

**HLA-A allele **(ME n = 73; TU n = 147)								
	ME	TU		ME	TU		ME	TU
					
A*010101	**0.137**	**0.088**	A*110101	0.048	0.030	A*3301	**0.055**	0.031
A*0102	0	0.003	A*2301	**0.082**	**0.078**	A*3303	0	0.01
A*0103	0	0.007	A*240201	**0.062**	0.037	A*33new*	0	0.003
A*020101	**0.178**	**0.173**	A*250101	0.007	0.007	A*3402	0.021	0.01
A*0202	0	0.034	A*2601	0.007	0.031	A*3601	0	0.003
A*0205	0	0.024	A*2902	0.041	**0.061**	A*6601	0.021	0.02
A*0212	0	0.003	A*3001	0.014	0.031	A*680101	0.021	0.031
A*0285	0	0.003	A*3002	**0.089**	0.044	A*680102	0.021	0.014
A*02new*	0	0.003	A*3004	0.007	0.003	A*680201	0.048	**0.054**
A*030101	0.034	**0.068**	A*3010	0	0.003	A*7401	0.014	0.003
A*030103	0	0.003	A*310102	0.021	0.024	A*8001	0	0.007
A*0302	0.041	0.017	A*3201	0.034	0.034			
								
**HLA-B allele **(ME n = 69; TU n = 148)								
	ME	TU		ME	TU		ME	TU
					
B*070201	0.022	0.041	B*3508	0	0.014	B*4901	**0.051**	0.027
B*0705,06	0.007	0.003	B*3701	0.007	0.007	B*5001	**0.073**	**0.139**
B*0801**	**0.087**	**0.064**	B*3801	0.014	0.020	B*5002	0.043	0.014
B*1302°°	0	0.017	B*390602	0	0.003	B*510101,02	0.043	0.054
B*1401	0.014	0.010	B*3910	0.007	0.007	B*5108	0	0.003
B*1402	0.036	**0.054**	B*3920	0	0.003	B*520101,02	0.007	0.024
B*1403	0	0.010	B*4001	0	0.010	B*5301^^	0.014	0.024
B*150101	0	0.014	B*4002	0.007	0.003	B*5303	0	0.003
B*1503	0.014	0.017	B*4101	0	0.017	B*53new*	0	0.003
B*1510	0	0.004	B*4102	**0.065**	0.020	B*5501	0	0.003
B*1516	0	0.017	B*4201	0	0.017	B*570101	0.014	0.003
B*151701	0	0.007	B*4202	0.007	0.007	B*5702	0	0.003
B*180101	**0.051**	0.023	B*4402	**0.094**	0.047	B*570302	0.007	0
B*2702	0.007	0.007	B*440301	**0.116**	**0.071**	B*5801	0.014	0.027
B*2703	0.007	0	B*440302	0.007	0	B*5802	0.007	0.003
B*2705,13^	0.021	0.010	B*4405	0	0.003	B*7301	0	0.007
B*350101,42	0.043	0.027	B*4427	0	0.007	B*7801	0.014	0
B*3502	0.007	0.017	B*4501	0.043	0.047			
B*3503	0.014	0.014	B*470101	0.007	0.003			
								
**HLA-Cw **allele (ME n = 63; TU n = 147)								
	ME	TU		ME	TU		ME	TU
					
Cw*0102	0	0.007	Cw*0602	**0.183**	**0.218**	Cw*140201	0.008	0.027
Cw*020202***	**0.095**	**0.054**	Cw*0701,06°	**0.183**	**0.129**	Cw*150201	0.016	0.034
Cw*0210	0.016	0.017	Cw*070201	0.032	**0.054**	Cw*150501,02	0.016	0.014
Cw*0302	0	0.010	Cw*0704,0711	0.016	0.010	Cw*1601	0.032	0.044
Cw*030301	0	0.003	Cw*0802	**0.056**	**0.058**	Cw*1602	0.016	0.007
Cw*030401, 02°°°	0	0.014	Cw*120201,02	0.008	0.024	Cw*160401	0	0.003
Cw*040101	**0.159**	**0.126**	Cw*120301	0.008	0.044	Cw*1701,02,03^^^	**0.071**	**0.051**
Cw*0501,03	**0.087**	0.048	Cw*12new*	0	0.003			

Alleles having a frequency > 5% are in bold. °Including Cw*070101,02; **Two samples in this group were assigned as B*080101; °°One sample in this group was assigned as B*130201; ^Two samples in this group were assigned as B*270502 (1ME, 1TU); ^^Two samples in this group were assigned as B*530101; ***One sample in this group was assigned as Cw*0202; °°°One sample in this group was assigned as Cw*0304; ^^^Cw*1703, af = 0.063 in ME.

Not discriminated alleles due to polymorphisms outside the assayed exons are listed separated by ",". Nomenclature was updated until the sixth digit; seventh and eighth digits were not included in the table. In case of ambiguous allele combinations, the most frequent allele combination was included in the analysis.

No statistically significant differences were identified in any comparison between the Tunisian and Moroccan cases.

**Table 2:** Most common (frequency > 2%) HLA-A/B, Cw/B and Cw/B two-locus haplotypes in TU

Haplotype	TUNISIANS					Present in:*
		
	HF	Dx100	D'	χ^2^	p	
**A/B**						
						
A*020101 B*5001	0.046	2.20	0.19	8.30	< 0.01	Caucasians, Hispanics, Tunisians^1^
A*020101 B*440201	0.037	2.90	0.74	38.00	< 0.001	Caucasians, Blacks
A*2301 B*5001	0.030	1.90	0.29	13.00	< 0.001	
A*6802 B*1402	0.024	2.10	0.41	50.00	< 0.001	Mixed
A*020101 B*510101	0.024	1.50	0.33	8.70	< 0.05	Caucasians, Hispanics, Blacks, Tunisians^1^
						
**Cw/B**						
Cw*0602 B*5001	0.122	9.10	0.84	120.00	< 0.001	Hispanics
Cw*0701 B*0801	0.047	3.80	0.69	65.00	< 0.001	Caucasians, Hispanics, Blacks, Tunisians^1^
Cw*0501 B*4402	0.041	3.90	0.87	22.00	< 0.001	Caucasians, Hispanics, Blacks, Tunisians^1^
Cw*0802 B*1402	0.037	3.40	0.66	120.00	< 0.001	Caucasians, Hispanics, Blacks, Tunisians^1^
Cw*0602 B*4501	0.034	2.40	0.65	22.00	< 0.001	Black
Cw*0702 B*070201	0.034	3.10	0.80	140.00	< 0.001	Caucasians, Blacks, Orientals
Cw*1502 B*510101	0.034	3.20	1.00	180.00	< 0.001	Caucasians, Hispanics, Tunisians^1^
Cw*0701 B*4901	0.027	2.30	0.99	54.00	< 0.001	Hispanics, Blacks, Tunisians^1^
Cw*0202 B*440301	0.027	2.20	0.33	32.00	< 0.001	
Cw*1601 B*440301	0.027	2.40	0.58	59.00	< 0.001	Caucasians, Hispanics, Tunisians^1^
Cw*1202 B*520101	0.024	2.30	1.00	30.00	< 0.001	Hispanics, Orientals, Tunisians^1^
Cw*1701,03 B*4102	0.020	1.90	1.00	110.00	< 0.001	Very common
Cw*040101 B*3501	0.020	1.70	0.70	28.00	< 0.001	Caucasians, Hispanics, Blacks, Tunisians^1^
Cw*040101 B*5301	0.020	1.70	0.81	33.00	< 0.001	Hispanics, Blacks
Cw*120301 B*3801	0.020	1.90	1.00	130.00	< 0.001	Caucasians, Hispanic, Tunisians^1^
						
**A/Cw**						
						
A*020101 Cw*0602	0.063	2.50	0.19	7.80	< 0.01	
A*2301 Cw*0602	0.036	1.90	0.31	7.70	< 0.01	
A*030101 Cw*0602	0.035	2.00	0.38	11.00	< 0.001	
A*010101 Cw*040101	0.034	2.30	0.30	18.00	< 0.001	Blacks
A*020101 Cw*0501	0.033	2.50	0.62	28.00	< 0.001	

In bold: haplotypes shared with ME. Other haplotypes (*in italics*) are shared but were not in linkage (p = ns) or were present at low frequency (< 1.5%) in ME.

HF: haplotype frequency; SD: standard deviation; Dx100: linkage disequilibrium multiplied by 100; D': relative linkage disequilibrium; ChiSQ: chi-square values

* According to the allele frequency worldwide population database [42,43].

**Table 3:** Most common HLA-A, -Cw, -B haplotypes in TU and ME.

Haplotypes	TU	ME
	
	HF SD	HF SD
**A0201 Cw0602 B5001**	0.054 ± 0.013	0.040 ± 0.019
A0201 **Cw0501, 03 B4402**	0.034 ± 0.011	(0.008 ± 0.107)
A2301 **Cw0602 B5001**	0.024 ± 0.009	-
A0201 Cw040101 B4403	(0.007 ± 0.005)	0.032 ± 0.016
A2301 Cw040101 B4403	(0.007 ± 0.005)	0.032 ± 0.014
A0101 **Cw0501, 03 B4402**	-	0.032 ± 0.017

Standard deviations (SD) have been approximated by 400 bootstrap replicates. In bold: shared haplotypes. In parenthesis: haplotype present at low frequency in one of the two populations.

By comparing in Tunisians the observed homozygosity F values with those expected under the Ewens-Watterson neutral model, a tendency toward balancing selection was observed, (observed F less than that expected under neutrality) although no significant difference in homozygosity was found (A locus: observed F: 0.068; expected F: 0.090; B locus: observed F: 0.048; expected F: 0.056; Cw locus: observed F: 0.101; expected F: 0.124).

### Single locus analysis

A total of 35 HLA-A, 51 HLA-B and 24 HLA-Cw alleles were observed in Tunisians. The most frequently detected alleles in Tunisians and Moroccans were: HLA-A*020101(TU:17.3%, ME:17.8%), A*010101 (TU:8.8%, ME:13.7%), A*2301 (TU:7.8%, ME:8.2%), HLA-B*5001(TU:13.9%, ME:7.3%), B*440301(TU:7.1%, ME:11.6%), B*0801(TU:6.4%, ME:8.7%), HLA-Cw*0602(TU:21.8%, ME:18.3%), Cw*040101(TU:12.6%, ME:15.9%) and Cw*07 group (TU:19.3%, ME:18.4%).

Noteworthy was the presence of rare alleles in Tunisians, as classified according to the National Marrow Donor Program [41], such as B*5303 (originally identified in Hispanics but present also in Africans and Caucasians) and A*0285 (Caucasian) and uncommon alleles present in Africans, such as A*3010 (identified in Moroccans), A*0103, A*3004, A*3601, A*8001, B*1403, B*4202, B*5702, Cw*0210 (present in Moroccans), A*030103, B*4405, B*4427, B*5108, B*7301, Cw*1604 (present in Caucasians), A*0212, B*390602, B*5303 (present in Hispanics) and B*3920 (present in Blacks). Although not significant, a lower frequency of B*44, with a wider number of alleles (some of which uncommon), was found in Tunisians compared to Moroccans (12.8% vs. 21.7%, p = ns), with a frequency similar to South Africans (Zulu 12%, Cameroon 8.7%). The HLA-B*50 allele frequencies were high in Tunisians (15.3%) as in Moroccans (11.6%) and in accordance with previously reported information about Tunisians of mixed origin (10%) [42]. Many allele groups showed a high variability (4–6 alleles, A*02, A*30, B*15, B*35, B*44). For instance, the HLA-B*15 group included the -B*1503 and -B*1510 alleles, that are present at high frequency in Sub-Saharan Africa. Overall, this data support the concept that Tunisians like other Maghrebian populations maintain a Northern African genetic identity which, however, includes traits derived from Sub-Saharan African [29] as well as Spanish/European influence [27]. The higher level of differentiation present in Moroccans, expressed by a reduced number of alleles compared to TU, can be a genetic demonstration of a higher isolation of this ethnic group.

### Extended haplotype analysis

One hundred and eighty different haplotypes were calculated in Tunisians. Data on haplotypes are shown in table 2 (2 loci) and table 3 (3 loci). The most common class I haplotypes were A*0201-B*5001-Cw*0602, shared by Tunisians (haplotype frequency; hf = 0.054) and Moroccans (hf = 0.040) and also present in Hispanics [43] (Table 3), and A*0201-B*4402-Cw*05, present in Hispanics, Africans and Caucasians [43,42]. The A*2301-B*5001-Cw*0602 was not found in other populations, like the A*2301-B*5001 2-loci association. Many Cw-B haplotypes, in strong linkage disequilibrium, are common haplotypes found in Hispanic, Caucasians, African and African American (Black) populations, and have been detected in other Tunisians [42]. Most haplotypes were shared between Tunisians and Moroccans (Table 2 and 3, in bold). Exceptions were represented by A*2301-B*5001and B4403-Cw0202, not found in other populations, and there were also not present (A*2301-B*5001) or present but in no significant linkage disequilibrium (B4403-Cw0202) in Moroccans.

### HLA class I frequencies in Tunisian patients with NPC

Of the 147 samples obtained from Tunisian subjects with NPC only 135 HLA-A, 135 HLA-B and 136 HLA-Cw locus typings could be carried out due to insufficient quantity or quality of the recovered genomic DNA (Figure 1). The age distribution of the cohort of NPC patients in this study well reflected the recognized bimodal age distribution of NPC in Tunisia. Indeed, the study included 32 patients with NPC that were 25 year old or younger corresponding to a frequency of 23% identical to the frequency reported for this age group by Daoud J *et al*. [44].

To compare our results with previously reported studies on NPC in Maghrebian populations, we first analyzed allele frequencies at a low resolution (two digits) level that more closely approximate the serological nomenclature. Simple two by two comparison tables identified HLA-B18, -B51 and -B57 to be associated with a predisposition to develop NPC (Table 4). On the contrary, HLA-B14 and Cw-08 were associated with a decreased risk of developing NPC. The haplotype frequency was strongly reduced in patients with NPC. At the phenotype level, 2 of 136 patients with NPC carried the HLA Cw*0802-B*1402 haplotype (1.47%) while 11 of 148 (7.4%) Tunisian controls carried this haplotype (Fisher's test p_2_-value < 0.001). With the exception of HLA-Cw-08, these findings specifically reproduced associations that had been previously described in Maghrebian populations [22,24] Thus, these results encouraged an in depth analysis of the HLA frequencies in patients with NPC from Tunisia at the high resolution level.

**Table 4:** Significant (Fisher's test p_2 _< 0.05) allele associations with NPC

HLA allele	NPC cases (%)	Control subjects (%)	OR	95% CI	P	Ref
Cw08	1.10 (3/272)	5.74 (17/296)	0.18	0.052 – 0.618	0.003	-
B14	0.74 (2/270)	7.43 (22/296)	0.09	0.021 – 0.388	0.0001	[22]
B14-Cw08	0.74 (2/270)	5.74 (17/296)	0.12	0.027 – 0.519	0.001	-
B51	11.11 (30/270)	5.74 (17/296)	1.98	1.067 – 3.679	0.025	[22]
B57	3.70 (10/270)	0.68(2/296)	5.48	1.190 – 25.224	0.017	[22]
B18	6.67 (18/270)	2.36 (7/296)	2.85	1.173 – 6.941	0.014	[24]

N.B. 136 typing could be performed for the HLA-Cw locus in patient with NPC while only 135 could be performed in the same patients for the HLA-A and -B loci.

### Extended haplotype analysis in Tunisian patients with NPC

The comparisons with the normal Tunisian and Moroccan populations were performed only for haplotypes found in Tunisians and/or patients with NPC (Table 5). Several associations were noted between NPC prevalence and HLA haplotypes. HLA-B*1801 was positively associated with the prevalence of NPC at the allele and haplotype level (specific NPC haplotype: A*020101-Cw*0701-B*180101, absent in Tunisians and Moroccans). The haplotype Cw*0501-B180101 was observed in Moroccans and, therefore, it may be less specific; HLA-B*570101 was also positively associated at the allele and haplotype level (specific NPC haplotype: A*010101-Cw*0602-B*570101, absent in Tunisians and Moroccans). Finally, HLA B*510101 was associated with an increased risk of developing NPC only at the allele but not the haplotype level.

**Table 5:** Comparison of the most common (frequency > 2%) HLA-A/B, Cw/B and A/Cw two-loci haplotypes in Tunisians, Tunisian NPC patients and in ME

Haplotype	TUNISIANS					NPC TUNISIANS					ME		
	
	HF	Dx100	D'	χ^2^	p	HF	Dx100	D'	χ^2^	p	HF	χ^2^	p
A/B													
													
A*020101 B*5001	0.045	2.20	0.20	8.9	< 0.01	0.044	2.70	0.33	16	< 0.001	0.029	3.50	NS
A*020101 B*440201	0.037	2.90	0.74	40.0	< 0.001	Not present					0.022	0.24	NS
A*2301 B*5001	0.030	2.00	0.29	14.0	< 0.001	(0.011)					Not present		
A*6802 B*1402	0.023	2.00	0.41	49.0	< 0.001	Not present					**(0.015)**		
A*020101 B*510101	0.020	1.10	0.25	5.1	< 0.05	(0.018)					(0.015)		
A*010101 B*570101	(0.003)					0.022	1.80	0.62	23	< 0.001	(0.015)		
A*020101 B*180101	(0.010)					0.030	1.80	0.33	10	< 0.01	0.022		NS
A*020101 B*440301	(0.013)					0.033	1.80	0.24	7.6	< 0.01	0.029		NS
A*2902 B*440301	(0.012)					0.021	1.60	0.29	15	< 0.001	Not present		
A*3402 B*0801	(0.006)					0.022	2.00	0.72	58	< 0.001	Not present		
													
Cw/B													
Cw*0701 B*0801	0.047	3.90	0.70	68.00	< 0.001	0.056	4.40	0.75	58	< 0.001	**0.089**	55.97	< 0.001
Cw*040101 B*4403	(0.016)					0.047	3.50	0.45	36	< 0.001	0.081	31.17	< 0.001
Cw*0602 B*5001	0.120	9.10	0.84	120.00	< 0.001	0.081	6.20	0.77	76	< 0.001	0.073	42.62	< 0.001
Cw*1701,03 B*4102	0.020	1.90	1.00	120.00	< 0.001	0.026	2.50	1.00	160	< 0.001	0.065	124.00	< 0.001
Cw*0701 B*4901	0.027	2.30	1.00	57.00	< 0.001	0.041	3.40	0.99	57	< 0.001	0.056	34.40	< 0.001
Cw*0802 B*1402	0.037	3.40	0.67	130.00	< 0.001	(0.007)					0.040	87.08	< 0.001
Cw*040101 B*3501	0.020	1.70	0.71	30.00	< 0.001	Not present					**0.040**	30.68	< 0.001
Cw*0501 B*4402	0.040	3.80	0.85	22.00	< 0.001	(0.018)	1.70	0.53	61	< 0.001	0.040	25.79	< 0.001
Cw*0501 B*1801	Not present					0.022	2.00	0.52	43	< 0.001	0.032	36.37	< 0.001
Cw*0602 B*4501	0.033	2.30	0.64	22.00	< 0.001	(0.011)	0.81	0.67	7.8	< 0.01	0.032	13.03	< 0.001
Cw*0702 B*070201	0.033	3.10	0.82	150.00	< 0.001	(0.018)	1.80	0.97	130	< 0.001	0.025	92.21	< 0.001
Cw*0202 B*440301	0.027	2.20	0.33	34.00	< 0.001	(0.012)					0.032		NS
Cw*1502 B*510101	0.033	3.20	1.00	180.00	< 0.001	0.044	4.10	1.00	140	< 0.001	(0.016)		
Cw*1601 B*440301	0.027	2.40	0.59	62.00	< 0.001	0.022	1.90	0.51	30	< 0.001	(0.008)		
Cw*1202 B*520101	0.023	2.30	1.00	30.00	< 0.001	0.030	2.90	0.89	220	< 0.001	Not present		
Cw*040101 B*5301	0.020	1.70	0.83	35.00	< 0.001	0.037	3.20	1.00	68	< 0.001	(0.008)		
Cw*120301 B*3801	0.020	1.90	1.00	140.00	< 0.001	(0.018)					Not present		
Cw*0602 B*1302	(0.017)					0.026	2.10	1.00	30	< 0.001	Not present		
Cw*020204 B*1503	(0.014)					0.033	3.20	1.00	280	< 0.001	(0.016)		
Cw*0701 B*180101	Not present					0.037	2.60	0.46	21	< 0.001	**(0.008)**		
Cw*0602 B*570101	(0.003)					0.033	2.70	1.00	40	< 0.001	(0.008)		
													
A/Cw													
A*020101 Cw*0602	0.061	2.40	0.18	7.60	< 0.01	0.023				NS	0.077	11.00	< 0.001
A*030101 Cw*0602	0.035	2.10	0.40	12.00	< 0.001	0.033	1.90	0.32	9.4	< 0.01	Not present		
A*010101 Cw*040101	0.033	2.20	0.29	17.00	< 0.001	(0.007)					Not present		
A*020101 Cw*0501,03	0.032	2.40	0.63	28.00	< 0.001	0.022	1.50	0.46	12	< 0.001	(0.012)		
A*2301 Cw*0602	0.032	1.60	0.27	6.50	< 0.05	(0.016)					Not present		
A*010101 Cw*0602	Not present					0.041	1.60	0.15	3.9	NS	0.026		NS
A*010101 Cw*0701	0.023				NS	0.037	1.60	0.14	4.2	NS	(0.016)		
A*020101 Cw*040101	(0.014)					0.037	1.40	0.13	3.3	NS	0.040		NS
A*020101 Cw*0701	0.023				NS	0.036				NS	0.020		NS
A*2902 Cw*1601	(0.010)					0.022	1.90	0.52	48	< 0.001	Not present		
A*3402 Cw*0701	(0.007)					0.022	1.70	0.69	20	< 0.001	(0.016)		

HF: haplotype frequency; SD: standard deviation; Dx100: linkage disequilibrium multiplied by 100; D': relative linkage disequilibrium; χ^2^: chi-square values In bold: alleles involved in NPC. In parenthesis: haplotypes present at low frequency (< 2%).

SBT identified several sub-haplotypes including an HLA-B*14 allele (Cw*0802-B*1402, A*6802-B*1402 and A*1101-Cw*0802-B*1402) that appeared to be responsible for the negative relationship between HLA-B14 and the prevalence of NPC in Tunisians. However, the most striking negative association was observed between the HLA-B*1402/Cw*0802 haplotype and the prevalence of NPC (HF = 0.007 in patient with NPC compared to a HF of 0.037 in Tunisians and 0.040 in Moroccans). In particular, all the HLA-Cw*08 alleles were identified by SBT as HLA-Cw*0802 while HLA-B14, split this haplotype into 3 sub-haplotypes in which HLA-B*1402 occurred 11 times (HF = 0.037) and HLA-B*1401 and B*1403 three times each (none of them seen in patients with NPC).

There was a trend toward an age distribution bias with the HLA class I loci associated with NPC predisposition occurring more frequently in the younger age bracket (<25 year-old patients). In particular, HLA-Cw*0602 was present at a frequency of 44% (14 of 32) in 25 year old or younger NPC patients compared to 16% (6 of 37) in patients who had NPC diagnosed after the age of 50 (Fisher test p2-value = 0.02). Similarly the HLA-B*5701/Cw*0602 haplotype was present in 12% of cases below age 25, 6% of cases between 25 and 50 and only 3% of cases above age 50. This trend was, however, not significant possibly because of to the relatively small number of cases. Interestingly, HLA-Cw*0602 and its extended haplotype HLA-B*5701/Cw*0602 were particularly frequent among young patients. Of 11 patients age 16 or younger, 7 (64%) carried the HLA-Cw*0602 allele and 4 the extended haplotype (37%) compared to a frequency of 25% and 4% respectively in the older patients (Fisher's exact test p_2_-value = 0.01 and 0.003 respectively). Interestingly, the same haplotype was totally absent in the same age group and it occurred only in one case among the 148 control subjects studied. Thus, it is possible that this haplotype may include a predisposition factor that may bear a particular influence on the penetrance of NPC before other environmental factors could contribute to the onset of NPC in later age groups.

## Discussion

NPC is among common cancers the one whose prevalence is most likely affected by a genetic predisposition that can be uncovered through population studies. This seems to particularly apply to the Southern Asian population repeatedly shown to be affected at high incidence. Although viral and environmental factors play an important role in the ethio-pathogenesis of NPC, genetic factors may also predispose as documented by several family and/or case-control studies [45,46]. The short arm of the human chromosome 6, is not the only genomic area that has been implicated in NPC predisposition as a large family study located a predisposition area in chromosome 3 [3]. However, many case-control studies pointed at HLA class I as a genomic region strongly associated with the prevalence of NPC in Southern Chinese [5-12,47-49] Such associations may bear two possible explanations; first the antigen presenting function of HLA molecules may be responsible for an altered effectiveness of the immune response against tumor associated antigens expressed by NPC in individuals bearing different HLA alleles [10]. This logical explanation is, however, challenged by the lack of candidate epitopic determinants associated to particular HLA class I alleles that clearly affect the natural history of NPC. In addition, most HLA class I associations reported in Asian populations indirectly support this concept since they are primarily associated with increased risk of developing NPC. This suggests that lack of antigen presenting capacity may result in reduced protection against the growth of NPC. For instance, the HLA-A*0207 allele association reported by Hildesheim et al. [10] in Taiwanese Chinese was explained by suggesting a reduced antigen presentation potential of this allele compared with other HLA-A*02 alleles present in the same population such as HLA-A*0201 which are not associated with higher prevalence of NPC. Similarly, the higher prevalence of HLA-A*0207 in Asians compared to Caucasians [10] may also explain the higher prevalence of the disease in Asian populations. However, associations of HLA phenotypes with increase prevalence of NPC are difficult to attribute to a single allele considering the high frequency of heterozygosity and the co-dominant expression of the three HLA class I loci aimed at broadening the antigen presenting repertoire of each individual.

An alternative explanation for the association between HLA and the prevalence of NPC attributes a broader significance of HLA class I genes as markers for other predisposition factors genetically controlled within a locus in close linkage disequilibrium [21]. For instance, even in Hildesheim's report, the extended haplotype HLA-A*0207/HLA-B*4601 was characterized by a higher odds ratio to develop NPC than the HLA-A*0207 allele alone suggesting a more complex genetic explanation for such association than the displacement by the HLA-A*0207 allele of other HLA class I alleles belonging to the HLA-A*02 family. In addition, our and others' experiences do not suggest strong differences in epitope binding and antigen presentation between HLA-A*0201 (the prototypic Caucasian HLA-A*02 allele) and HLA-A*0207. These alleles differ only by one amino acid substitution from a Tyrosine to a Cysteine in position 99 within the binding groove of the HLA molecule. This substitution does not seem to significantly affect binding and presentation of canonical HLA-A*0201-associated epitopes [50-52] as well as EBV-associated epitopes [53]. Finally, lack of antigen presentation capacity by a specific HLA class I allele should be compensated by other alleles expressed by the same individual; therefore, favouring homozygosity of the unfavourable allele in affected individuals. However, most reports do not suggest higher homozygosity values in high prevalence populations suggesting lack of balancing selection. Thus, dominant genetic traits directly related to oncogenesis may play a more important role than HLA class I alleles which are co-dominantly expressed.

Maghrebs have been less exhaustively studied. However, serological analyses observed positive associations between NPC and HLA class I alleles. These include increased frequency of HLA-B5 in Algerians (38.2% *vs *24.4%) [22], HLA-B13 in Tunisians (15.5% vs 4) [23] and HLA-B18 in Moroccans (relative risk = 4.14) [24]. In addition, negative associations have been reported such as HLA-Aw33, -B14 in Algerians with NPC. However, after correction for the number of specificities tested, these differences were not statistically significant and were not pursued further. HLA-A*3301 and HLA-B*1402 have subsequently been recognized to belong to the same ancestral haplotype [25] suggesting that this haplotype may harbor more relevance to NPC than previously recognized. This impression was supported also by the identification of a negative association between HLA-A23 (known to belong to the extended HLA-A*2301/HLA-B*1403 haplotype [26]) and the prevalence of NPC [23]. This observation converges toward the relevance of HLA-B14 related haplotypes which may bear protective effects against NPC in North Africans. Moreover, Dardari R et al. [24] reported a significantly lower frequency of the HLA-A9 serologic family alleles in NPC patients compared with controls. Since the latter study did not provide molecular resolution, we can only speculate that the reduced frequency of HLA-A9 reflected a lower frequency of the HLA-A*2301/HLA-B*1403 haplotype since HLA-A23 is part of the HLA-A9 serological family. Thus, previous studies in Maghrebians suggested that HLA-B14 and HLA-A alleles linked to the HLA-B14 serological family may be under-represented in patients with NPC compared with normal controls. Conversely, HLA-B5, -B13 and -B18 could be associated with increased risk of NPC.

Low resolution (two digits level) analysis of allele frequency in our study quite remarkably and specifically confirmed previous positive associations described between NPC and HLA class I alleles in Berber populations. Both HLA-B*18 and -B*51 (the latter representing a split of the serologically-defined HLA-B5 family) were confirmed by our study (Table 4). In addition, a novel association was observed between HLA-B-57 (belonging to the HLA-B17 serological family) and the prevalence of NPC. Only the positive association observed in Tunisians between HLA-B13 and NPC prevalence was not reproduced by this study [23]. Negative associations were also identified by this study of which only HLA-B*14 (OR = 0.09, Fisher's test p-2 value = 0.0001) strongly reproduced previous findings while no association was noted with HLA-A*23 nor HLA-A*33. Interestingly, however, another strong negative association was noted between HLA-Cw*0802 and NPC (OR = 0.18, Fisher's test p-2 value = 0.003). This is of particular significance because both HLA-B*1402 and HLA-Cw*0802 belong to the same ancestral haplotype which was present not only in Tunisians but also Moroccan Berbers but practically absent in Tunisian patients with NPC (OR = 0.12, Fisher's test p-2 value = 0.001). This strong negative association suggests that this haplotype may bear a locus in strong linkage disequilibrium with these HLA alleles with dominant protective effects against the onset of NPC in North Africans such as a tumor suppressor gene with stronger stability in this phenotype. Since protective alleles have also been described in Asian populations (such as HLA-A11) [13], it could be postulated that some HLA alleles may bear immunological functions facilitating the immune surveillance against NPC. However, there is no known functional similarity between alleles described in Chinese and North Africans to support this hypothesis suggesting that other factors may bear on this predisposition. Indeed, in Asian populations, micro-satellite analyses within the short arm of Chromosome 6 identified associations between putative loci and NPC prevalence that are stronger than those with HLA class I alleles [12]. Hu C-C *et al*. [54] using high-resolution micro satellite mapping recently described a carcinoma-susceptibility locus located in a segment of Chromosome 6p21.3 containing the class I loci. Similarly, Nh SP *et al*. [55] concluded that an HLA-related recessive mutation is responsible for the prevalence of NPC in Chinese. Finally, Tian W et al. [56] identified in the Southern Chinese Han population a positive association between a predisposition to develop NPC and exon 5 of the MICA-STR encoded within the HLA class I region. Moreover, a recent meta-analysis of chromosomal aberrations associated with NPC [57] identified a small region in Chromosome 6p21.2-p23 that is frequently amplified in NPC and contains various candidate genes that may be responsible for NPC [58]. Thus, our findings suggest that there are haplotypes within or close to HLA class I region, particularly the HLA-B and -C loci that may bear genetic characteristics favoring or protecting against the onset of NPC in Tunisian populations at risk. This information may help identify individuals belonging to different ethnic groups whose genetic risk to develop NPC could be marked by HLA class I haplotypes. These prototypic phenotypes could be scanned with novel high throughput technologies covering genomic areas in high linkage disequilibrium with such haplotypes. Since the analysis of the Tunisian control population did not identify differences between Tunisians and Moroccans, considering that a common Berber substratum is shared by North African countries [59-63], it is possible that our findings could be relevant to the other Northern African populations justifying the extension of future studies to Moroccans and Algerians [24].

Some of the alleles discussed by this study have been associated with other pathologies. HLA-B*1403 is the predominant allele in patients affected by ankylosing spondylitis from West Africa, where HLA-B*27 is uncommon [64]. HLA-B*51 and HLA-B*15 alleles have been associated with Behcet disease [65], B*44, B*51, B*57, B*15 with HPV infection and protection/susceptibility to in cervical carcinoma [66]. The present study, may offer novel insight on the molecular genetics of this region that may influence disease, although obvious relationships between the information presented and it causality is still missing.
